# Enhanced Uptake and Phototoxicity of C_60_@albumin Hybrids by Folate Bioconjugation

**DOI:** 10.3390/nano12193501

**Published:** 2022-10-06

**Authors:** Andrea Cantelli, Marco Malferrari, Edoardo Jun Mattioli, Alessia Marconi, Giulia Mirra, Alice Soldà, Tainah Dorina Marforio, Francesco Zerbetto, Stefania Rapino, Matteo Di Giosia, Matteo Calvaresi

**Affiliations:** Dipartimento di Chimica “Giacomo Ciamician”, Alma Mater Studiorum-Università di Bologna, Via Francesco Selmi 2, 40126 Bologna, Italy

**Keywords:** C_60_, fullerenes, human serum albumin, folate, photodynamic therapy, phototheranostic platform

## Abstract

Fullerenes are considered excellent photosensitizers, being highly suitable for photodynamic therapy (PDT). A lack of water solubility and low biocompatibility are, in many instances, still hampering the full exploitation of their potential in nanomedicine. Here, we used human serum albumin (HSA) to disperse fullerenes by binding up to five fullerene cages inside the hydrophobic cavities. Albumin was bioconjugated with folic acid to specifically address the folate receptors that are usually overexpressed in several solid tumors. Concurrently, tetramethylrhodamine isothiocyanate, TRITC, a tag for imaging, was conjugated to C_60_@HSA in order to build an effective phototheranostic platform. The in vitro experiments demonstrated that: (i) HSA disperses C_60_ molecules in a physiological environment, (ii) HSA, upon C_60_ binding, maintains its biological identity and biocompatibility, (iii) the C_60_@HSA complex shows a significant visible-light-induced production of reactive oxygen species, and (iv) folate bioconjugation improves both the internalization and the PDT-induced phototoxicity of the C_60_@HSA complex in HeLa cells.

## 1. Introduction

Fullerenes have attracted much attention as potential PDT photosensitizers for use as antimicrobial and anticancer therapies [1,2,3,4,5]. Compared with the photosensitizers commonly used in PDT [6,7], fullerenes have several advantages that include:

(i) A higher photostability and lower photobleaching than conventional dyes;

(ii) An ability to generate reactive oxygen species (ROS) following both type I and type II photophysical mechanisms, while conventional dyes primarily follow the type II process; 

(iii) A high efficacy of ROS production, with a quantum yield of ^1^O_2_ generation that is close to unity.

However, the use of fullerenes in PDT still has significant practical limitations due to the lack of water solubility and their low biocompatibility [1,2,3,4,5]. Another key component of the efficacious use of fullerenes in PDT is their monomolecular dispersion in solution. Indeed, aggregation is a well-known element that causes fullerenes to be inactive as photosensitizers [8,9,10]. The characteristics of the fullerene dispersion affect its photophysical and photochemical properties at the molecular level [11]. The crucial intermediate ^3^C_60_*, which produces ROS, interacting with the ground state of molecular oxygen, is extremely environment-sensitive [8,9,10,11]. Aggregation significantly quenches or shortens the lifespan of the long-lived triplet excited state, which lowers the efficiency of the ROS generation [11]. In addition, aggregation phenomena also decrease the surface area of C_60_ that comes into contact with oxygen and actively produces ROS [8,9,10,11].

Supramolecular hosts can be used to efficiently disperse fullerenes in aqueous media without any modification of their molecular structures. In particular, fullerenes can be dispersed as single molecules in physiological contexts, avoiding aggregation, by using biomolecules [4], such as proteins and peptides [12,13,14,15,16,17,18,19,20,21]. Proteins play a role similar to that of “Trojan Horses” [22], since they (i) can disperse fullerenes, hiding the cages inside their cavities, and (ii) regulate their cellular uptake, maintaining their biological identity.

Albumins are ideal protein carriers for therapeutic agents and photosensitizers because of their unique properties, such as biocompatibility, biodegradability, their absence of toxicity, non-immunogenicity and long blood circulation [23,24,25,26,27,28]. Human serum albumin (HSA) is a single-chain protein with a molecular weight of 66.7 kDa, composed of 585 amino acids [23,24,25,26,27,28]. HSA is water-soluble and is the most abundant protein in human blood plasma, with a half-life of approximately 21 days [23,24,25,26,27,28]. HSA is the natural carrier of hydrophobic molecules in the blood. Albumins have already demonstrated their ability to interact with fullerene derivatives [29,30,31,32,33,34,35,36,37,38,39,40,41,42,43,44]. HSA exhibits passive tumor targeting due to its enhanced permeability and retention (EPR) effect. As a result of the leaky vasculature and lack of lymphatic drainage in tumor tissues, HSA can extravasate and accumulate in the tumor tissue, reaching high intratumor concentrations [23,24,25,26,27,28]. Additionally, two albumin-binding proteins, gp60 (a 60 kDa albumin-binding glycoprotein) and the albumin-binding protein SPARC (secreted protein acidic and rich in cysteine), also facilitate the absorption and retention of albumin in cancer cells via active targeting [23,24,25,26,27,28]. Albumin can also be functionalized using targeting ligands to specifically address the overexpressed receptors on the surface of cancer cells, i.e., EGFR [45], folate receptors (FR) [46] or interleukin-13 alpha 2 receptor (IL13Rα2) [47]. In particular, folate receptors are glycoproteins (35–40 kDa), which are widely overexpressed on the surfaces of numerous cancer cells, such as the ovarian, triple-negative breast and lung [46,48]. As a result, the folate receptors represent a natural choice for the development of cancer-targeted delivery systems [46,48].

Here, we demonstrated that HSA can bind and monomolecularly disperse C_60_ in physiological conditions. The resulting C_60_@HSA complex is strongly photoactive, producing large numbers of ROS (following both type I and type II photophysical mechanisms) when exposed to visible light. In addition, since the protein platform offers a variety of chemical groups for functionalization, the performance of the C_60_@HSA biohybrids can also be improved by attaching i) folate molecules to selectively target the folate receptors, enhancing cellular uptake, or ii) imaging tags, such as TRITC, to build phototheranostic platforms [20,26,49]. The PDT-killing activity of C_60_@HSA, and especially that of C_60_@HSA-folate, can be observed in HeLa cells upon light irradiation, while the hybrids are completely biocompatible in dark conditions.

## 2. Materials and Methods

### 2.1. Synthesis and Characterization of the Biohybrids

#### 2.1.1. Materials

Human serum albumin (HSA) (Cat. No. A3782), folic acid (FA) (Cat. No. F7876), *N*-hydroxysulfosuccinimide sodium salt (NHS) (Cat. No. 56485), N-(3-dimethylaminopropyl)-*N*′-ethylcarbodiimide hydrochloride (EDC), 10-Acetyl-3,7-dihydroxyphenoxazine (Amplex Red) (Cat. No. 90101), type VI-A peroxidase from horseradish lyophilized powder (HRP) (Cat. no. P6782), hydrogen peroxide solution 30% (*w/w*) (Cat. No. 31642-M), 9,10-anthracenediylbis (methylene) dimalonic acid (ABMDMA) (Cat. No. 75068), deuterium oxide (Cat. No. 151882-100G), tetramethylrhodamine isothiocyanate mixed isomers (TRITC, Cat. No. 87918), Amicon Ultra centrifugal filters (MWCO 30 kDa, Millipore UFC503024) (Cat. No. Z677892-24EA), dimethyl sulfoxide (DMSO) (Cat. No. 472301), sodium chloride (Cat. No. S9888-M), potassium phosphate monobasic (Cat. No. P0662-M), sodium phosphate dibasic (Cat. No. S0876), potassium chloride (Cat. No. P3911M), sodium bicarbonate (Cat. No. 31437-M), sodium carbonate (Cat. No. 223530) and MWCO 14 kDa dialysis tubing cellulose membrane (Cat. No. D9652) were purchased from Sigma Aldrich (Merck). Milli-Q water was used for the preparation of all the aqueous solutions.

#### 2.1.2. C_60_@HSA Synthesis and Purification

A total of 33 mg of HSA was dissolved in 5 mL of PBS. A total of 7.2 mg of C_60_ powder was added, and the solution was sonicated for two hours in an ice bath at 45% of the maximum amplitude with a tip sonicator Hielscher Ultrasonic Processor UP200St, equipped with a sonotrode S26d7. To remove the insoluble C_60_ aggregates, the resulting turbid brown mixture was centrifugated for 10 min at 10,000 rpm at room temperature, and the orange/yellow supernatant was collected. The recovered solution was then extensively dialyzed against 400 mL of PBS three times using cellulose membrane dialysis tubes with a 14 kDa cut-off.

#### 2.1.3. Amplex Red Assay for Peroxides Quantification

The ability to generate peroxides in solution upon irradiation with visible light was evaluated using an Amplex Red fluorometric assay. HRP catalyzes the reaction between the colorless nonfluorescent Amplex Red and peroxides to form the colored and fluorescent resorufin. A total of 10 μL of Amplex Red 50 mM in DMSO was added to 1 mL of phosphate buffer of 50 mM, pH 7.4. Then, 10 μL of HRP of 0.4 mg/mL in PBS was added to the Amplex Red solution to obtain the final working solution. A total of 90 μL of the solutions under investigation, in phosphate buffer saline of 50 mM, pH 7.4, was irradiated for 10 min with visible light on 96-well microtiter plates (cold white LED, Valex, 30 W lamp at a distance of 30 cm from the plate, irradiation power density on the cell plate = 2.4 mW/cm^2^, measured with the photo-radiometer Delta Ohm LP 471 RAD). A total of 10 μL of Amplex Red working solution was added to each sample (and to the corresponding references, kept in the dark) immediately after irradiation. After 30 min of incubation at room temperature, the fluorescence of the samples was recorded at 590 nm, using 560 nm as the excitation wavelength. A calibration curve generated using standard solutions of H_2_O_2_ was used to convert the fluorescence signal to the peroxide concentration generated upon irradiation. Fluorescence measurements were carried out using a Perkin Elmer EnSpire^®^ (Waltham, MA, USA) Multimode Plate Reader.

#### 2.1.4. The ^1^O_2_ NIR Fluorometric Detection

Singlet oxygen emission spectra were recorded with an Edinburgh FLS920 spectrofluorometer equipped with a Ge detector for emissions in the NIR spectral region. The sample buffer was exchanged for deuterated PBS (prepared in D_2_O) using Amicon Ultra-0.5 centrifugal filter units (MWCO 30 kDa) for three cycles, restoring the initial volume of the samples. Emissions were recorded in the 1100–1500 nm spectral range in aerobic conditions, with an excitation wavelength of 514 nm. A correction of the emission spectra for the detector sensitivity was performed.

#### 2.1.5. C_60_@HSA-FA Synthesis and Purification

A total of 5 mg of folic acid was dissolved in 0.5 mL of DMSO. The solution was mixed with vortex for one hour until complete dissolution was achieved. A total of 6 mg of EDC and 2 mg of NHS were then added. The solution was mixed for a further 5 h in the dark. A total of 200 μL of this solution was added dropwise to the C_60_@HSA solution under vigorous stirring (1000 rpm). At the end of the addition, the solution was kept under moderate stirring overnight in the dark. The day after, the solution was filtered with a 200 nm RC syringe filter. The solution was then dialyzed against 400 mL of PBS to remove unconjugated folate molecules and reaction coproducts. The dialysis cycles were stopped when no trace of folate was observed in the UV–Vis spectra of the dialysis waters.

#### 2.1.6. C_60_@HSA-FA-TRITC Synthesis and Purification

TRITC was dissolved in DMSO to obtain a 5 mM stock solution. A total of 20 μL of this solution was then added dropwise to 380 μL of the C_60_@HSA and C_60_@HSA-FA solutions (both 50 μM in 100 mM sodium carbonate buffer, pH 9). The mixture was then incubated overnight at 25 °C under continuous shaking at 700 rpm (ThermoMixer HC, S8012-0000; STARLAB, Hamburg, Germany). To remove the unconjugated TRITC, the bioconjugates were dialyzed using 10 mM sodium carbonate buffer (pH 9) and using a cellulose membrane. The UV–Vis spectra of the dialysate were obtained to monitor the purification process. To remove the alkaline buffer, the last dialysis cycle was performed against PBS.

#### 2.1.7. Characterization of the HSA Bioconjugates

The absorption spectra were recorded using a Cary60 UV–Vis spectrophotometer (Agilent).

Mass spectra were acquired using ACQUITY UPLC equipped with a BEH300 C4 column (2.5 µm, 4.6 × 150 mm, Waters Corporation, Milford, Worcester, MA, USA) and coupled with an ESI-QTOF mass spectrometer (Waters Corporation, Milford, Worcester, MA, USA). The concentration of the protein bioconjugates was 100 μM. The samples were injected at a flow rate of 0.2 mL/min and eluted by a gradient elution (phase A: 0.1% formic acid in H_2_O, phase B: 0.1% formic acid in ACN; 0 min 80%A 20%B, 30 min 30%A 70%B, 31 min 80%A 20%B, 50 min 80%A 20%B; column temperature 40 °C). Positive-ion ESI mass spectra were acquired using a capillary voltage of 3 kV, a sample cone of 50 V and a desolvation gas flow of 800 l/h. The source and desolvation temperatures were 150 and 350 °C, respectively. All spectra were acquired in the range of *m/z* 50–2000. Raw data were background-subtracted and deconvoluted using Unidec software in the range of *m/z* 1000–2000.

Agarose gel electrophoresis was performed using an Owl Easycast B-Series Horizontal Gel Systems Model B2. The electrophoresis was performed using a 0.5 cm thick 1% agarose gel. PBS was used as the sample buffer and Tris-glycine at pH 7.4 was used as the running buffer. A total of 15 μg of protein conjugates, containing 20% *v/v* of glycerol, were loaded into each well. The run was performed by applying a potential of 100 V.

Dynamic light scattering (DLS) measurements were performed using a Malvern Instruments DLS ZetaSizer Nano-ZS. DLS measurements were carried out using a concentration of the protein bioconjugates of 5μM in PBS at room temperature. 

The stability of the bioconjugates was evaluated over 3 h (the incubation time used for the in vitro experiments) at 37 °C in PBS by UV–Vis measurements following the absorbance of the fullerene diagnostic band at 341 nm. The concentration of all the protein bioconjugates was 5 μM.

### 2.2. Computational Details

#### 2.2.1. Docking

The crystal structure of HSA (PDB ID: 1N5U) was downloaded from the Protein Data Bank (PDB) and used for the docking calculations. Docking models were obtained using the PatchDock algorithm, which computes the shape complementarity between two entities (ligand and receptor), minimizing the number of steric clashes. PatchDock (i) assigns concave, convex or flat patches to the ligand and receptor surfaces; (ii) matches concave–convex/flat–flat and generates a set of candidate transformations; and (iii) ranks the different poses, using a scoring function that takes in account the shape complementarity and the atomic desolvation energy of the complex (the most important terms in the case of fullerenes). The first 15 docking poses were selected for the minimization, equilibration and MD trajectory production stage. Using these 15 MD trajectories, the binding affinity was calculated using the MM/GBSA protocol, and the best five binding sites were determined. 

#### 2.2.2. Minimization and MD Simulations

The Amber ff14SB force field was used to model the HSA. THe C_60_ carbon atoms were modelled as uncharged Lennard-Jones particles using sp^2^ carbon parameters taken from the GAFF force field. A total of 500 steps of steepest descent minimization, followed by an additional 9500 steps of conjugate gradient minimization, were performed with PMEMD for the 15 selected poses. The minimized structures were used as starting points for the equilibration process. Periodic boundary conditions (PBC) and the particle mesh Ewald summation were used throughout (with a cut-off radius of 10.0 Å). H-atoms were considered using the SHAKE algorithm and a time step of 2 fs was applied in all the MD runs. The MD simulations were carried out using an explicit solvent (TIP3P water model). Sodium counterions were included to precisely neutralize the charge of the system. Then, we applied 10 ns of heating to 298 K using an NPT ensemble and temperature coupling according to Andersen in order to equilibrate the system. This was followed by 100 ns of the MD trajectories. Snapshot structures were saved into individual trajectory files every 1000 time steps, i.e., every 2 ps of the molecular dynamics.

#### 2.2.3. Molecular Mechanics/Generalized Born Surface Area (MM/GBSA) Analysis

A total of 1000 frames were extracted from the MD simulations and used for the MM/GBSA analysis. An infinite cut-off was used for all the interactions. The electrostatic contribution to the solvation free energy was calculated using the generalized Born (GB) model, as implemented in MMPBSA.py. The nonpolar contribution to the solvation free energy was determined using solvent-accessible, surface-area-dependent terms. 

### 2.3. Cellular Experiments

#### 2.3.1. Internalization Studies of C_60_@HSA-TRITC and C_60_@HSA-FA-TRITC

HeLa cells (ATCC reference number: CCL-2) were cultured at 37 °C and in 5% CO_2_ in DMEM (Dulbecco’s Modified Eagle Medium; ThermoFisher Scientific, Waltham, MA, USA, 21969035) supplemented with 10% fetal bovine serum (South America), 2 mM L-glutamine, 50 U/mL penicillin and 50 µg/mL streptomycin. For the C_60_@HSA-TRITC and C_60_@HSA-FA-TRITC internalization experiments, 50.000 HeLa cells were plated on each 3.5 cm-diameter Petri dish and grown overnight in the incubator, and the cell density was evaluated as previously reported [50]. The day after the cell plating, the growing medium was substituted for a fresh one, supplemented with 3.5 µM C_60_@HSA-TRITC and C_60_@HSA-FA-TRITC. For the control samples (i.e., HeLa cells not incubated with any C_60_@HSA derivatives), the growing medium was replaced with a fresh one without further supplementation. After 3 and 18 h of incubation with the C_60_@HSA-TRITC and C_60_@HSA-FA-TRITC, the medium was discharged, and the samples were washed with PBS supplemented with calcium and magnesium (ThermoFisher product number: 14040091) and measured in the presence of Hanks Balanced Salt Solution (HBSS; Sigma Aldrich product number H8264). Each sample was washed just before the acquisition of the fluorescence and phase contrast data. For each sample, 3 fields of view with a 20× magnification were used for both the phase contrast and fluorescence (Nikon Texas Red HYQ cubic filter (λ excitation = 532–587 nm, λ emission = 608–683 nm)) images, for which a Nikon Eclipse Ti inverted microscope was employed. The internalization of the C_60_@HSA derivatives was quantified by determining the average pixel intensity of the manually selected region of interests (ROIs) in each fluorescence image using Fiji (ImageJ) software [51]. The ROIs were drawn by contouring the region of each cell in a fluorescence image using a phase contrast image as the reference. For each image, the basal mean fluorescence was determined as the average of 3 ROIs drawn in the regions of the image with no cells, while the mean basal fluorescence was subtracted to obtain the mean fluorescence value of each cell ROI. The statistical analysis was performed with Origin 2019b software by two-tailed t-test analysis (considering populations with different variances) and ANOVA two-way analysis.

#### 2.3.2. Cytotoxicity and Photodynamic Activity of C_60_@HSA and C_60_@HSA-FA

The photodynamic activity of C_60_@HSA and C_60_@HSA-FA was evaluated on HeLa cells, which were plated at a density of 2·10^4^ cells per well on 48-well plates. Incubation for 3 h (37 °C, 5% CO_2_) in dark conditions was performed using different concentrations of C_60_@HSA and C_60_@HSA-FA. Before light treatment, the cells were washed using PBS. Samples were irradiated for 10 min with a white light source kept at a distance of 30 cm from the cell-plate. The irradiation power density of the cell plate was 2.4 mW/cm^2^ (measured with the photo-radiometer Delta Ohm LP 471 RAD). In parallel, the control samples were left in the same environmental conditions but in the absence of any exposure to light (i.e., dark conditions). After 24 h of further incubation, upon photo-irradiation, the cell viability was quantified by MTT assay.

## 3. Results and Discussion

**Figure 1:**
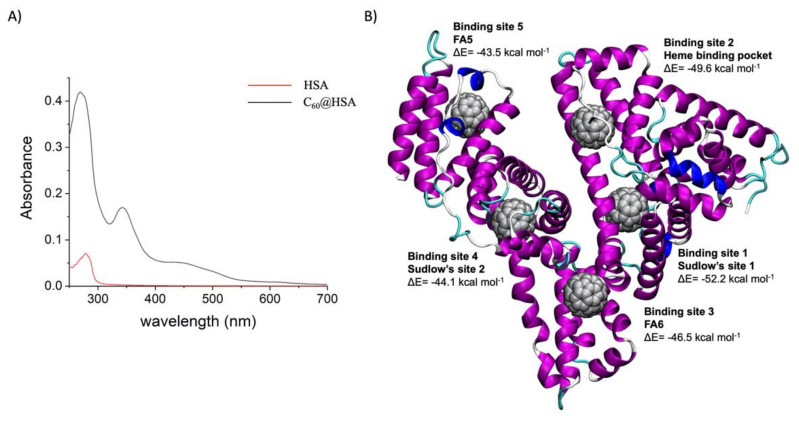
(**A**) UV–visible spectra of C_60_@HSA (black line) and HSA (red line) in PBS. In Figure S1, the UV–visible spectrum of C_60_ in toluene is depicted. (**B**) The most probable fullerene binding pocket in HSA, identified by a docking algorithm followed by molecular dynamics simulations.

C_60_@HSA was synthesized by adding C_60_ powder to a HSA solution contained in an Eppendorf tube. The solution was ultrasonicated and centrifuged to remove the excess C_60_. A brown solution was obtained by collecting the supernatant. C_60_ is totally insoluble in water. The UV–Vis spectrum of the solution (Figure 1A) clearly showed the diagnostic band of the fullerene, centered at 341 nm, indicating its presence in the water solution and proving the formation of the C_60_@HSA complex.

The electrophoretic characterization of C_60_@HSA clearly confirmed the encapsulation of C_60_ in the protein (Figure S2). Considering the initial HSA concentration and the molar extinction coefficients of C_60_ (see SI for the determination of the binding stoichiometry), an average number of 4.3 fullerene molecules were identified, which were bound to a single HSA. This stoichiometry is not surprising, since albumin has seven hydrophobic fatty acid binding sites (FA1–FA7). Using a docking methodology used to recognize the sites of interaction between proteins and carbon nanomaterials [52,53,54,55,56,57,58,59,60], we docked C_60_ in the HSA crystal structure to identify the most probable C_60_ binding site in the HSA. We identified 15 possible C_60_ binding sites (see Figure S3). Starting with these poses, MD simulations were carried out, followed by MM/GBSA analysis of the trajectories, to obtain the binding energy of C_60_ in these pockets. The five most probable binding sites (Figure 1B) were identified: the Sudlow site 1 (FA7), the heme binding pocket (FA1), the albumin-binding side FA6, the Sudlow site 2 (FA3,4) and the albumin-binding side FA5. The fullerene cage consistently showed a strong shape complementarity with these HSA pockets (Figures S4–S8). Fullerene binding requires the existence of a hydrophobic cavity in the protein in order to accommodate the fullerene cage. A direct consequence is that van der Waals interactions are the most crucial term for the recognition and binding of C_60_ to HSA. Nonpolar solvation (the hydrophobic effect) assists with the binding, while polar solvation typically works against it. The contribution of each amino acid to the binding is determined by the decomposition analysis (fingerprint analysis) of the overall binding energy (see Figures S2–S6). The amino acids with the largest binding contributions are aromatic amino acids (Tyr 138, Tyr150, Tyr161, His 242), characterized by π-π interactions with C_60_ [61], followed by hydrophobic amino acids (Leu238, Leu327, Leu347, Pro384, Leu387, Ile388, Ile513, Leu516, Val555), interacting with the fullerene cage via vdW and the hydrophobic effect [61], and charged amino acids (Glu141, Lys351), interacting with C_60_ via surfactant-like interactions [61].

ROS formation in biological samples can be evaluated using different bioanalytical approaches [62,63]. Two different pathways exist for the production of ROS upon light absorption by C_60_ (See Scheme S1 in SI). When irradiated with the appropriate wavelength, C_60_ is excited to singlet excited states (S_n_). An intersystem crossing (ISC), which is formally forbidden, although, in C_60_, the quantum yield is close to the unit, leads C_60_ to a T_1_ excited state (^3^C_60_*). This state is characterized by a long lifespan, and its interaction with O_2_ can generate reactive oxygen species (ROS) through two alternative pathways. 

In the type I mechanism (electron transfer mechanism), ^3^C_60_* acquires a hydrogen atom or an electron to form a radical, which further reacts with water or molecular oxygen, leading to the production of different ROS, such as superoxide anions, hydroxyl radicals, and hydrogen peroxide.

In the type II mechanism (energy transfer mechanism), an energy transfer between ^3^C_60_* and molecular oxygen occurs, forming a highly reactive singlet oxygen excited state (^1^O_2_).

**Figure 2:**
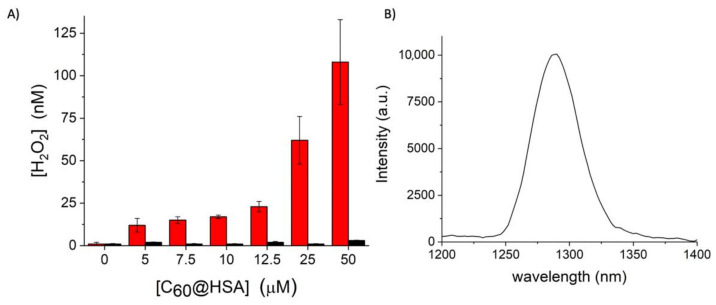
(**A**) Generation of peroxides during visible light irradiation (red histograms) and in the dark (black histograms) using different concentrations of C_60_@HSA. (**B**) Emission spectra of singlet oxygen generated upon the excitation of C_60_@HSA.

We used the Amplex Red assay to measure the generation of peroxides upon irradiation with visible light (Figure 2A) [64,65,66] to determine if type I mechanism is active. The number of peroxides produced is proportional to the C_60_@HSA concentration. The lack of a plateau in the formation of peroxides when the C_60_ concentration increases, as is frequently observed in the case of aggregation phenomena, confirms the monomolecular dispersion of the C_60_ molecules in the solution. The C_60_@HSA hybrids produce peroxides only when exposed to visible light, as was confirmed by control experiments using the same C_60_@HSA solutions, which were kept in the dark (Figure 2A).

The ability of C_60_@HSA to produce singlet oxygen (^1^O_2_) in aqueous media upon visible light irradiation was also demonstrated by measuring the near-IR emission of ^1^O_2_ centered at 1270 nm (Figure 2B) [65], indicating that the type II mechanism is also active. 

These data clearly demonstrate that the C_60_@HSA complex follows both the type I and type II photophysical mechanisms of ROS production.

**Figure 3:**
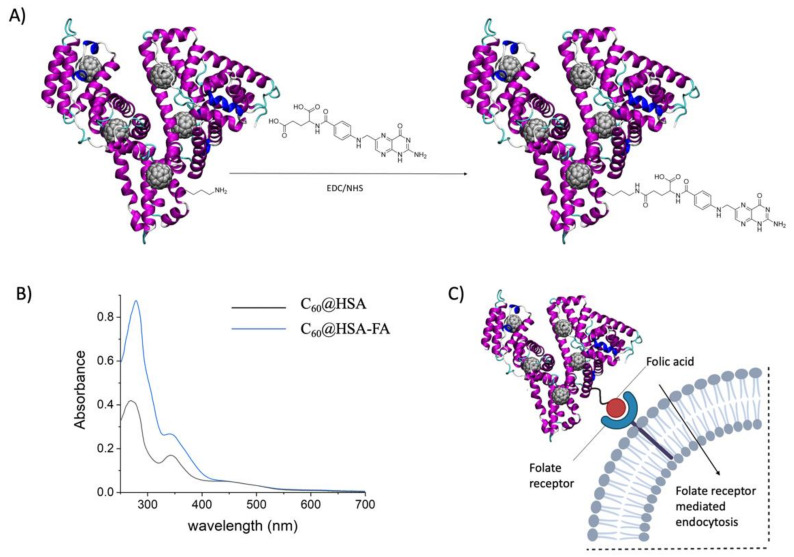
(**A**) Bioconjugation of folic acid to C_60_@HSA. (**B**) UV–visible spectra of C_60_@HSA (black line) and C_60_@HS-FA (blue line) in PBS. In Figure S9, the UV–visible spectrum of FA in PBS. (**C**) Folate-receptor-mediated endocytosis.

The protein platform can be functionalized using targeting ligands for an effective drug delivery and to target specifically overexpressed receptors. Folic acid (FA) is one of the best options for the targeted killing of cancer cells. Due to the high folate demand, the folate receptor alpha (FRα), a membrane-bound protein, is extensively expressed on the surfaces of many human cancer cells. In contrast, the expression of FRα is minimal in normal cells. FA is recognized by FRα even when it is covalently attached to a biomolecule or a nanoparticle [67,68,69,70]. The decoration of albumins [67,68] and albumin nanoparticles with folate is a common strategy for targeting FRα [69,70], which enables an enhanced targeted delivery to FRα-overexpressing cancer cells through FRα-mediated endocytosis (Figure 3). 

We conjugated folic acid (FA) on the surfaces of the C_60_@HSA complexes via an EDC/NHS cross-coupling reaction between the carboxylic acid moiety of the folic acid and the amine groups of HSA (Figure 3A).

Normalizing the UV–Vis spectra of C_60_@HSA and C_60_@HSA-FA (Figure 3B) at 450 nm (where only absorption by C_60_ occurs), it is possible to estimate the number of FA molecules conjugated per HSA, considering their absorbance at 363 nm. Based on the molar extinction coefficient, at 363 nm of FA (6197 M^−1^cm^−1^) [71], an average of 7 FA molecules is conjugated to each C_60_@HSA complex. The mass spectra of the C_60_@HSA-FA prove the successful completion of the bioconjugation process (Figure S10).

**Figure 4:**
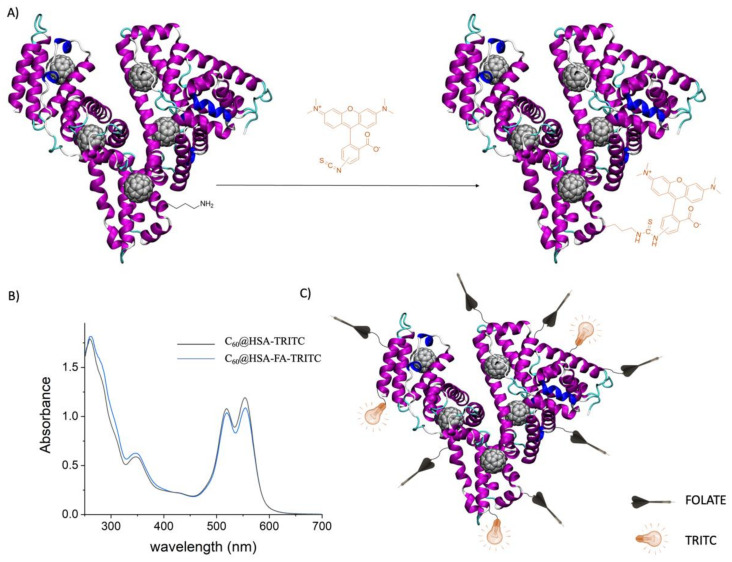
(**A**) Bioconjugation of TRITC to C_60_@HSA/C_60_@HSA-FA. (**B**) UV–visible spectra of C_60_@HSA-TRITC (black line) and C_60_@HSA-FA-TRITC (blue line) in PBS. In Figure S11, the UV–visible spectrum of TRITC in PBS. (**C**) Creation of an C_60_@HSA-based theranostic platform by protein conjugation of the targeting agents (FA) and imaging tags (TRITC).

HSA contains 59 lysine residues and 1 terminal amine group. Therefore, there are 60 amine groups that have potential as modification sites. Because we exploited only seven sites for the FA bioconjugation on average, the opportunity to also conjugate imaging tags to the targeted protein platform, in order to create a real phototheranostic platform, remained. We decided to conjugate tetramethylrhodamine isothiocyanate (TRITC) to the C_60_@HSA-FA complex. The isothiocyanate reactive group of the dye reacts with the amine groups of HSA, forming thiourea bond linkages (Figure 4A). Considering the molar extinction coefficients of TRITC at 554 nm, equating to 95,000 M^−1^cm^−1^, approximately 2.5 TRITC molecules are conjugated to C_60_@HSA/C_60_@HSA-FA (Figure 4B). 

Exploiting the fluorescent properties of TRITC, the effective conjugation of the dye to C_60_@HSA-FA can be detected by agarose gel electrophoresis (Figure S12).

We therefore armed HSA with C_60_ molecules (C_60_@HSA), applied the C_60_@HSA complex to the cancer cells by folate conjugation (C_60_@HSA-FA) and provided the system with imaging properties by linking TRITC to the protein (C_60_@HSA-FA-TRITC). The result was an effective phototheranostic platform (Figure 4C).

The DLS analysis of the bioconjugates (Figure S13) demonstrated that the presence of the hydrophobic fullerenes induces the oligomerization of HSA. We already observed this phenomenon when lysozyme was used as the dispersing agent for C_70_ [20]. Proteins tend to cover the exposed part of the bound fullerenes, which is strongly hydrophobic, with their hydrophobic patches inducing non-covalent oligomerization processes. In addition, it is well-known that HSA has a strong tendency to form well-defined, non-covalent aggregates [72], such as dimers, tetramers or higher oligomers. DLS sets the size of the HSA as ~7 nm, corresponding to the monomeric structure of the protein, while providing a well-defined size of ~14 nm for C_60_@HSA. The dimension of the aggregate reflects the formation of a dimer [72,73]. Agarose gel electrophoresis confirmed that the oligomerization process is non-covalent (Figure S12). The conjugation of folate and TRITC to the C_60_@HSA causes, as expected, an increase in the hydrodynamic radius of the oligomers (~20 nm for C_60_@HSA-FA and ~23 nm for C_60_@HSA-FA-TRITC). Measurements of the stability (Figure S14) of the C_60_@HSA, C_60_@HSA-FA and C_60_@HSA-FS-TRITC complexes over time, in physiologically relevant conditions (PBS), showed that the encapsulation of C_60_ in HSA solves the problem of the poor stability of C_60_ in a saline environment. Such a problem is present, for instance, when C_60_ is dispersed with γ-cyclodextrins or is in the form of nanoparticles (nC_60_) that rapidly precipitate as soon as NaCl is added [74].

**Figure 5:**
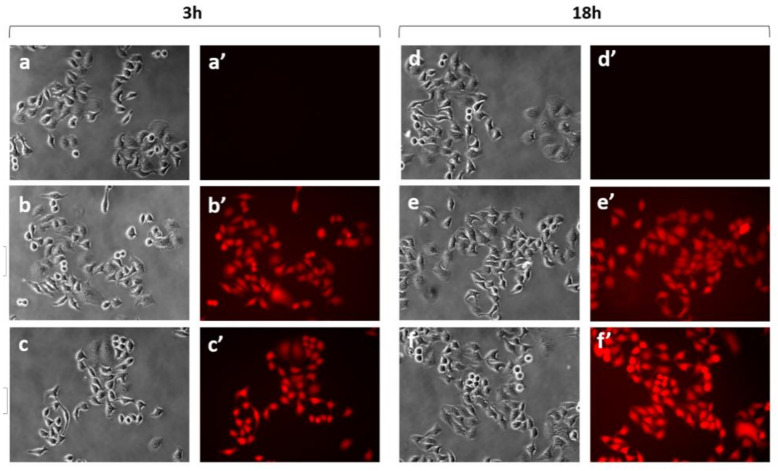
Representative phase contrast (**a**–**f**) and TRITC fluorescence (**a′**–**f′**) images measured on the control HeLa samples after 3 h (**a**,**a′**) and 18 h (**d**,**d′**); HeLa cells incubated with C_60_@HSA-TRITC after 3 h (**b**,**b′**) and 18 h (**e**,**e′**); C_60_@HSA-FA-TRITC after 3 h (**c**,**c′**) and 18 h (**f**,**f′**).

To assess the uptake and the PDT performances of C_60_@HSA and C_60_@HSA-FA, HeLa cells were selected, since they overexpress folic acid receptors on their surfaces [75]. The internalization of C_60_@HSA-TRITC and C_60_@HSA-FA-TRITC by HeLa cells was quantitatively evaluated by employing the fluorescence signals of the TRITC molecules. Representative phase contrast and fluorescence images of the HeLa cells following incubation with HSA derivatives are shown in Figure 5. 

The HeLa cells incubated with both C_60_@HSA-TRITC and C_60_@HSA-FA-TRITC showed cytoplasmatic red fluorescence after 3 h of incubation, demonstrating that the internalization of C_60_@HSA took place both in the presence and in the absence of folate derivatization. Control experiments performed on the HeLa cells, which were not incubated with any C_60_@HSA-TRITC derivatives, did not reveal any relevant fluorescence signals. 

**Figure 6:**
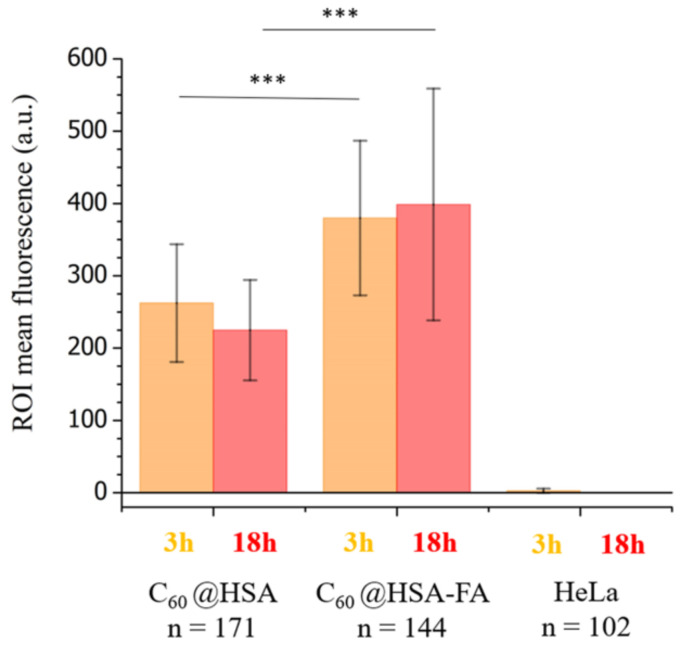
Average mean cell fluorescence signals after 3 h (orange) and 18 h (red) of incubation, determined for single HeLa cells using the fluorescence images (see Materials and Methods for details on the acquisition parameters and on the signal treatment); error bars are standard deviations. For C_60_@HSA-TRITC and C_60_@HSA-FA-TRITC and the HeLa control samples, the following numbers of ROIs were considered: C_60_@HSA-TRITC, n = 171; C_60_@HSA-FA-TRITC, n = 144; HeLa, n = 102. Statistical significance was evaluated with a two-tailed t-test using different variances in the populations (***, *p* < 0.001).

The quantification of the mean fluorescence intensity of each cell after 3 and 18 h of incubation is reported in Figure 6.

The significance of the fluorescence increase was tested through the t-test and ANOVA analysis (Figure 6 and Table S1, see the Materials and Methods section for details on the statistical analysis). Both the t-test and ANOVA statistical analysis showed that folate significantly increases the fluorescence signals after both 3 and 18 h of incubation. These results proved that folate increases the internalization of C_60_@HSA derivatives by Hela cells. It is important that the increase in the internalization of C_60_@HSA after 3 h of incubation was also maintained after a longer incubation time (18 h). Folate promotes both the early and late internalization of C_60_@HSA derivatives, without any relevant activation of the cell clearance mechanisms that could induce a lower C_60_@HSA concentration in the cell cytoplasm over a longer timescale.

**Figure 7:**
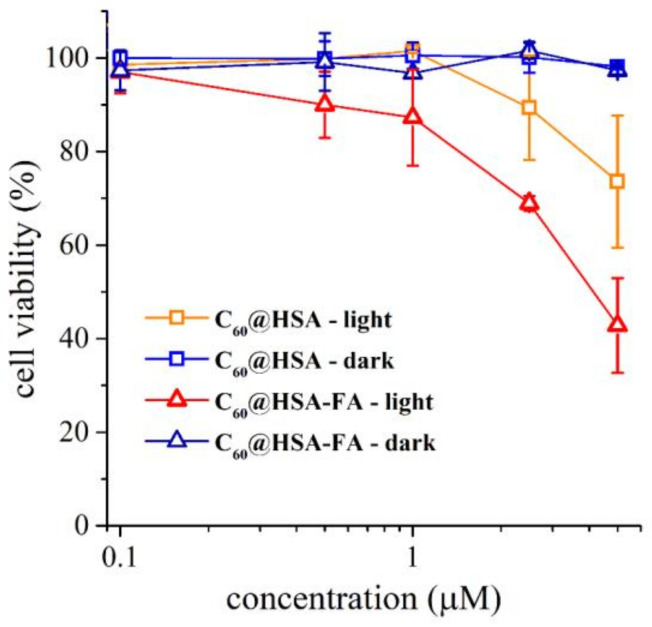
Phototoxicity of C_60_@HSA and C_60_@HSA-FA in the 0.1–5 µM range. Percentage of cell viability for irradiated and not irradiated HeLa cells incubated with C_60_@HSA and C_60_@HSA-FA. C_60_@HSA: irradiated, orange squares; not irradiated, electric blue squares. C_60_@HSA-FA: irradiated, red triangles; not irradiated, dark blue triangles. Each value represents the average ± 1 standard deviation of at least 2 independent measures.

Based on the results of the internalization experiments, we studied the photoinduced killing activity of C_60_@HSA and C_60_@HSA-FA against the HeLa cells. Cell viability curves obtained by incubating the cells with a concentration of HSA derivatives in the range of 0.1–5 µM are reported in Figure 7. No dark toxicity was observed for C_60_@HSA and C_60_@HSA-FA across the whole range of concentrations. This aspect is crucial, because the use of HSA to disperse C_60_ overcomes the two most important limitations on the use of fullerenes in nanomedicine: the lack of water solubility and their low biocompatibility.

On the contrary, upon irradiation, the presence of C_60_@HSA and C_60_@HSA-FA caused a marked reduction in the viability of the HeLa cells in a dose-dependent manner. In agreement with the uptake studies, showing a higher internalization of HSA due to folate derivatization, it appears that (i) the photokilling activity of C_60_@HSA-FA occurs at a lower concentration of the hybrids, and (ii) the PDT efficiency of C_60_@HSA-FA is approximately twice that of C_60_@HSA.

Our results confirmed that the bioconjugation of targeting moieties (FAs) on the protein platform increases the cellular uptake of the adduct through a ligand-receptor-based mechanism.

## 4. Conclusions

The use of HSA to disperse C_60_ bypasses the two most important limitations that have hampered the full exploitation of fullerene in nanomedicine, namely, the lack of water solubility and low biocompatibility.

The resulting C_60_@HSA complex shows a significant visible-light-induced production of ROS, following both type I and type II photophysical mechanisms. 

Exploiting the C_60_@HSA platform, which offers different chemical groups for chemical functionalization, the performances of the C_60_@HSA biohybrids were improved by coupling with:

(i) Folate molecules to target selectively folate receptors which, in turn, enhances the cellular uptake of the adduct by the cancer cells and promotes the internalization of the fullerene;

(ii) TRITC molecules to provide imaging properties to the adduct. 

In the in vitro experiments, using HeLa cells, a cell line that overexpress folic acid receptors on the surfaces, it was shown that folate bioconjugation enhances both the internalization and the PDT-induced phototoxicity of the C_60_@HSA complex.

The C_60_@HSA platform can be additionally improved by using, for example, light-harvesting antennae in order to enhance the ROS production [76,77,78] and to extend the PDT activity of the fullerene further into the red/NIR [76,77,78,79], or by attaching other imaging tags to create targeted multimodal theranostic platforms [80].

## Data Availability

All data in this study can be requested from the corresponding authors (stefania.rapino3@unibo.it, S.R.; matteo.digiosia2@unibo.it, M.D.G.; and matteo.calvaresi3@unibo.it, M.C.).

## Supplementary Materials

The following supporting information can be downloaded at: https://www.mdpi.com/article/10.3390/nano12193501/s1, Figure S1: Normalized UV-visible spectrum of C_60_ in toluene; Figures S2: Agarose gel electrophoresis of HSA and C_60_@HSA; Figure S3: Possible binding site of C_60_ in HSA; Figures S4-S8: Interaction between HSA and C_60_ bound in the binding sites 1–5; Scheme S1: Jablonski’s diagram showing Type I and type II mechanism of ROS generation. Figure S9: Normalized UV-visible spectrum of FA in PBS; Figure S10: Mass spectra of HSA before and after conjugation with FA; Figure S11: Normalized UV-visible spectrum of TRITC in PBS; Figure S12: Agarose gel electrophoresis of HSA, C_60_@HSA, C_60_@HSA-FA and C_60_@HSA-FA-TRITC. Figure S13: Particle number size distribution of HSA, C_60_@HSA, C_60_@HSA-FA and C_60_@HSA; Figure S14: Evolution of the stability of C_60_@HSA, C_60_@HSA-FA and C_60_@HSA as a function of time, in PBS; Table S1: ANOVA two-way analysis. Scheme S1. Jablonski’s diagram showing Type I and type II mechanism of ROS generation.

## References

[1] Markovic Z., Todorovic-Markovic B., Kleut D., Nikolic N., Vranjes-Djuric S., Misirkic M., Vucicevic L., Janjetovic K., Isakovic A., Harhaji L. (2007). The mechanism of cell-damaging reactive oxygen generation by colloidal fullerenes. Biomaterials.

[2] Markovic Z., Trajkovic V. (2008). Biomedical potential of the reactive oxygen species generation and quenching by fullerenes (C60). Biomaterials.

[3] Sharma S.K., Chiang L.Y., Hamblin M.R. (2011). Photodynamic therapy with fullerenes in vivo: Reality or a dream?. Nanomedicine.

[4] Antoku D., Sugikawa K., Ikeda A. (2019). Photodynamic Activity of Fullerene Derivatives Solubilized in Water by Natural-Product-Based Solubilizing Agents. Chem.—A Eur. J..

[5] Hamblin M.R. (2018). Fullerenes as photosensitizers in photodynamic therapy: Pros and cons. Photochem. Photobiol. Sci..

[6] Ormond A.B., Freeman H.S. (2013). Dye sensitizers for photodynamic therapy. Materials.

[7] Lan M., Zhao S., Liu W., Lee C.S., Zhang W., Wang P. (2019). Photosensitizers for Photodynamic Therapy. Adv. Healthc. Mater..

[8] Hotze E.M., Labille J., Alvarez P., Wiesner M.R. (2008). Mechanisms of photochemistry and reactive oxygen production by fullerene suspensions in water. Environ. Sci. Technol..

[9] Zhao B., He Y.-Y., Chignell C.F., Yin J.-J., Andley U., Roberts J.E. (2009). Difference in Phototoxicity of Cyclodextrin Complexed Fullerene [(γ-CyD)2/C60] and Its Aggregated Derivatives toward Human Lens Epithelial Cells. Chem. Res. Toxicol..

[10] Chae S.R., Badireddy A.R., Farner Budarz J., Lin S., Xiao Y., Therezien M., Wiesner M.R. (2010). Heterogeneities in fullerene nanoparticle aggregates affecting reactivity, bioactivity, and transport. ACS Nano.

[11] Guldi D.M., Prato M. (2000). Excited-state properties of C60 fullerene derivatives. Acc. Chem. Res..

[12] Calvaresi M., Arnesano F., Bonacchi S., Bottoni A., Calò V., Conte S., Falini G., Fermani S., Losacco M., Montalti M. (2014). C60@Lysozyme: Direct observation by nuclear magnetic resonance of a 1:1 fullerene protein adduct. ACS Nano.

[13] Di Giosia M., Bomans P.H.H., Bottoni A., Cantelli A., Falini G., Franchi P., Guarracino G., Friedrich H., Lucarini M., Paolucci F. (2018). Proteins as supramolecular hosts for C60: A true solution of C60 in water. Nanoscale.

[14] Soldà A., Cantelli A., Di Giosia M., Montalti M., Zerbetto F., Rapino S., Calvaresi M. (2017). C60@lysozyme: A new photosensitizing agent for photodynamic therapy. J. Mater. Chem. B.

[15] Di Giosia M., Nicolini F., Ferrazzano L., Soldà A., Valle F., Cantelli A., Marforio T.D., Bottoni A., Zerbetto F., Montalti M. (2019). Stable and Biocompatible Monodispersion of C 60 in Water by Peptides. Bioconjug. Chem..

[16] Kim K.-H., Ko D.-K., Kim Y.-T., Kim N.H., Paul J., Zhang S.-Q., Murray C.B., Acharya R., DeGrado W.F., Kim Y.H. (2016). Protein-directed self-assembly of a fullerene crystal. Nat. Commun..

[17] Vance S.J., Desai V., Smith B.O., Kennedy M.W., Cooper A. (2016). Aqueous solubilization of C60 fullerene by natural protein surfactants, latherin and ranaspumin-2. Biophys. Chem..

[18] Di Giosia M., Valle F., Cantelli A., Bottoni A., Zerbetto F., Calvaresi M. (2018). C60 bioconjugation with proteins: Towards a palette of carriers for all pH ranges. Materials.

[19] Liutkus M., López-Andarias A., Mejías S.H., López-Andarias J., Gil-Carton D., Feixas F., Osuna S., Matsuda W., Sakurai T., Seki S. (2020). Protein-directed crystalline 2D fullerene assemblies. Nanoscale.

[20] Di Giosia M., Soldà A., Seeger M., Cantelli A., Arnesano F., Nardella M.I., Mangini V., Valle F., Montalti M., Zerbetto F. (2021). A Bio-Conjugated Fullerene as a Subcellular-Targeted and Multifaceted Phototheranostic Agent. Adv. Funct. Mater..

[21] Di Costanzo L., Geremia S. (2020). Atomic details of carbon-based nanomolecules interacting with proteins. Molecules.

[22] Di Giosia M., Zerbetto F., Calvaresi M. (2021). Incorporation of Molecular Nanoparticles Inside Proteins: The Trojan Horse Approach in Theranostics. Accounts Mater. Res..

[23] Kratz F. (2014). A clinical update of using albumin as a drug vehicle—A commentary. J. Control. Release.

[24] Hoogenboezem E.N., Duvall C.L. (2018). Harnessing albumin as a carrier for cancer therapies. Adv. Drug Deliv. Rev..

[25] Chen Q., Liu Z. (2016). Albumin Carriers for Cancer Theranostics: A Conventional Platform with New Promise. Adv. Mater..

[26] Cantelli A., Malferrari M., Soldà A., Simonetti G., Forni S., Toscanella E., Mattioli E.J., Zerbetto F., Zanelli A., Di Giosia M. (2021). Human Serum Albumin–Oligothiophene Bioconjugate: A Phototheranostic Platform for Localized Killing of Cancer Cells by Precise Light Activation. JACS Au.

[27] Tao C., Chuah Y.J., Xu C., Wang D.A. (2019). Albumin conjugates and assemblies as versatile bio-functional additives and carriers for biomedical applications. J. Mater. Chem. B.

[28] Teixeira S., Carvalho M.A., Castanheira E.M.S. (2022). Functionalized Liposome and Albumin-Based Systems as Carriers for Poorly Water-Soluble Anticancer Drugs: An Updated Review. Biomedicines.

[29] Rozhkov S.P., Goryunov A.S., Sukhanova G.A., Borisova A.G., Rozhkova N.N., Andrievsky G.V. (2003). Protein interaction with hydrated C60 fullerene in aqueous solutions. Biochem. Biophys. Res. Commun..

[30] Belgorodsky B., Fadeev L., Ittah V., Benyamini H., Zelner S., Huppert D., Kotlyar A.B., Gozin M. (2005). Formation and Characterization of Stable Human Serum Albumin−Tris-malonic Acid [C60]Fullerene Complex. Bioconjug. Chem..

[31] Zhen M., Zheng J., Ye L., Li S., Jin C., Li K., Qiu D., Han H., Shu C., Yang Y. (2012). Maximizing the relaxivity of Gd-complex by synergistic effect of HSA and carboxylfullerene. ACS Appl. Mater. Interfaces.

[32] Giełdoń A., Witt M.M., Gajewicz A., Puzyn T. (2017). Rapid insight into C60 influence on biological functions of proteins. Struct. Chem..

[33] Li J., Jiang L., Zhu X. (2015). Computational studies of the binding mechanisms of fullerenes to human serum albumin. J. Mol. Model..

[34] Leonis G., Avramopoulos A., Papavasileiou K.D., Reis H., Steinbrecher T., Papadopoulos M.G. (2015). A Comprehensive Computational Study of the Interaction between Human Serum Albumin and Fullerenes. J. Phys. Chem. B.

[35] Krumkacheva O.A., Timofeev I.O., Politanskaya L.V., Polienko Y.F., Tretyakov E.V., Rogozhnikova O.Y., Trukhin D.V., Tormyshev V.M., Chubarov A.S., Bagryanskaya E.G. (2019). Triplet Fullerenes as Prospective Spin Labels for Nanoscale Distance Measurements by Pulsed Dipolar EPR Spectroscopy. Angew. Chemie.

[36] Serda M., Szewczyk G., Krzysztyńska-Kuleta O., Korzuch J., Dulski M., Musioł R., Sarna T. (2020). Developing [60]Fullerene Nanomaterials for Better Photodynamic Treatment of Non-Melanoma Skin Cancers. ACS Biomater. Sci. Eng..

[37] Zhang X., Shu C., Xie L., Wang C., Zhang Y., Xiang J., Li L., Tang Y. (2007). Protein Conformation Changes Induced by a Novel Organophosphate-Containing Water-Soluble Derivative of a C60 Fullerene Nanoparticle. J. Phys. Chem. C.

[38] Belgorodsky B., Fadeev L., Kolsenik J., Gozin M. (2006). Formation of a soluble stable complex between pristine C 60-fullerene and a native blood protein. ChemBioChem.

[39] Benyamini H., Shulman-Peleg A., Wolfson H.J., Belgorodsky B., Fadeev L., Gozin M. (2006). Interaction of C60-Fullerene and Carboxyfullerene with Proteins: Docking and Binding Site Alignment. Bioconjugate Chem..

[40] Zhang M.F., Xu Z.Q., Ge Y.S., Jiang F.L., Liu Y. (2012). Binding of fullerol to human serum albumin: Spectroscopic and electrochemical approach. J. Photochem. Photobiol. B Biol..

[41] Qu X., Komatsu T., Sato T., Glatter O., Horinouchi H., Kobayashi K., Tsuchida E. (2008). Structure, Photophysical Property, and Cytotoxicity of Human Serum Albumin Complexed with Tris(dicarboxymethylene)[60]fullerene. Bioconjug. Chem..

[42] Abdulmalik A., Hibah A., Zainy B.M., Makoto A., Daisuke I., Masaki O., Kaneto U., Fumitoshi H. (2013). Preparation of soluble stable C60/human serum albumin nanoparticles via cyclodextrin complexation and their reactive oxygen production characteristics. Life Sci..

[43] Li S., Zhao X., Mo Y., Cummings P.T., Heller W.T. (2013). Human serum albumin interactions with C60 fullerene studied by spectroscopy, small-angle neutron scattering, and molecular dynamics simulations. J. Nanoparticle Res..

[44] Song M., Liu S., Yin J., Wang H. (2011). Interaction of human serum album and C 60 aggregates in solution. Int. J. Mol. Sci..

[45] Ulfo L., Costantini P.E., Di Giosia M., Danielli A., Calvaresi M. (2022). EGFR-Targeted Photodynamic Therapy. Pharmaceutics.

[46] Fernández M., Javaid F., Chudasama V. (2018). Advances in targeting the folate receptor in the treatment/imaging of cancers. Chem. Sci..

[47] Kang Y.J., Holley C.K., Abidian M.R., Madhankumar A.B., Connor J., Majd S. (2021). Tumor Targeted Delivery of an Anti-Cancer Therapeutic: An In Vitro and In Vivo Evaluation. Adv. Healthc. Mater..

[48] Scaranti M., Cojocaru E., Banerjee S., Banerji U. (2020). Exploiting the folate receptor α in oncology. Nat. Rev. Clin. Oncol..

[49] Zhou J., Li J., Ding X., Liu J., Luo Z., Liu Y., Ran Q., Cai K. (2015). Multifunctional Fe2O3@PPy-PEG nanocomposite for combination cancer therapy with MR imaging. Nanotechnology.

[50] Bartolini L., Malferrari M., Lugli F., Zerbetto F., Paolucci F., Pelicci P.G., Albonetti C., Rapino S. (2018). Interaction of Single Cells with 2D Organic Monolayers: A Scanning Electrochemical Microscopy Study. ChemElectroChem.

[51] Schneider C.A., Rasband W.S., Eliceiri K.W. (2012). NIH Image to ImageJ: 25 years of image analysis. Nat. Methods.

[52] Calvaresi M., Zerbetto F. (2010). Baiting proteins with C60. ACS Nano.

[53] Calvaresi M., Zerbetto F. (2011). Fullerene sorting proteins. Nanoscale.

[54] Ahmed L., Rasulev B., Kar S., Krupa P., Mozolewska M.A., Leszczynski J. (2017). Inhibitors or toxins? Large library target-specific screening of fullerene-based nanoparticles for drug design purpose. Nanoscale.

[55] Calvaresi M., Furini S., Domene C., Bottoni A., Zerbetto F. (2015). Blocking the passage: C60 geometrically clogs K+ channels. ACS Nano.

[56] Di Giosia M., Marforio T.D., Cantelli A., Valle F., Zerbetto F., Su Q., Wang H., Calvaresi M. (2020). Inhibition of α-chymotrypsin by pristine single-wall carbon nanotubes: Clogging up the active site. J. Colloid Interface Sci..

[57] Bologna F., Mattioli E.J., Bottoni A., Zerbetto F., Calvaresi M. (2018). Interactions between Endohedral Metallofullerenes and Proteins: The Gd@C 60 –Lysozyme Model. ACS Omega.

[58] Di Giosia M., Valle F., Cantelli A., Bottoni A., Zerbetto F., Fasoli E., Calvaresi M. (2019). High-throughput virtual screening to rationally design protein—Carbon nanotube interactions. Identification and preparation of stable water dispersions of protein—Carbon nanotube hybrids and efficient design of new functional materials. Carbon N. Y..

[59] Berto M., Di Giosia M., Giordani M., Sensi M., Valle F., Alessandrini A., Menozzi C., Cantelli A., Gazzadi G.C., Zerbetto F. (2021). Green Fabrication of (6,5)Carbon Nanotube/Protein Transistor Endowed with Specific Recognition. Adv. Electron. Mater..

[60] Marforio T.D., Mattioli E.J., Zerbetto F., Calvaresi M. (2022). Fullerenes against COVID-19: Repurposing C60 and C70 to Clog the Active Site of SARS-CoV-2 Protease. Molecules.

[61] Marforio T.D., Calza A., Mattioli E.J., Zerbetto F., Calvaresi M. (2021). Dissecting the supramolecular dispersion of fullerenes by proteins/peptides: Amino acid ranking and driving forces for binding to c60. Int. J. Mol. Sci..

[62] Dikalov S.I., Harrison D.G. (2012). Methods for Detection of Mitochondrial and Cellular Reactive Oxygen Species. Antioxid. Redox Signal..

[63] Malferrari M., Ghelli A., Roggiani F., Valenti G., Paolucci F., Rugolo M., Rapino S. (2018). Reactive Oxygen Species Produced by Mutated Mitochondrial Respiratory Chains of Entire Cells Monitored Using Modified Microelectrodes. ChemElectroChem.

[64] Cantelli A., Piro F., Pecchini P., Di Giosia M., Danielli A., Calvaresi M. (2020). Concanavalin A-Rose Bengal bioconjugate for targeted Gram-negative antimicrobial photodynamic therapy. J. Photochem. Photobiol. B Biol..

[65] Bortot B., Apollonio M., Baj G., Andolfi L., Zupin L., Crovella S., di Giosia M., Cantelli A., Saporetti R., Ulfo L. (2022). Advanced photodynamic therapy with an engineered M13 phage targeting EGFR: Mitochondrial localization and autophagy induction in ovarian cancer cell lines. Free Radic. Biol. Med..

[66] Ulfo L., Cantelli A., Petrosino A., Costantini P.E., Nigro M., Starinieri F., Turrini E., Zadran S.K., Zuccheri G., Saporetti R. (2022). Orthogonal nanoarchitectonics of M13 phage for receptor targeted anticancer photodynamic therapy. Nanoscale.

[67] Butzbach K., Rasse-Suriani F.A.O., Gonzalez M.M., Cabrerizo F.M., Epe B. (2016). Albumin–Folate Conjugates for Drug-targeting in Photodynamic Therapy. Photochem. Photobiol..

[68] Dosio F., Arpicco S., Stella B., Brusa P., Cattel L. (2009). Folate-mediated targeting of albumin conjugates of paclitaxel obtained through a heterogeneous phase system. Int. J. Pharm..

[69] Dubey R.D., Alam N., Saneja A., Khare V., Kumar A., Vaidh S., Mahajan G., Sharma P.R., Singh S.K., Mondhe D.M. (2015). Development and evaluation of folate functionalized albumin nanoparticles for targeted delivery of gemcitabine. Int. J. Pharm..

[70] Akbarian A., Ebtekar M., Pakravan N., Hassan Z.M. (2020). Folate receptor alpha targeted delivery of artemether to breast cancer cells with folate-decorated human serum albumin nanoparticles. Int. J. Biol. Macromol..

[71] Kranz D.M., Patrick T.A., Brigle K.E., Spinella M.J., Roy E.J. (1995). Conjugates of folate and anti-T-cell-receptor antibodies specifically target folate-receptor-positive tumor cells for lysis. Proc. Natl. Acad. Sci. USA.

[72] Chubarov A., Spitsyna A., Krumkacheva O., Mitin D., Suvorov D., Tormyshev V., Fedin M., Bowman M.K., Bagryanskaya E. (2021). Reversible dimerization of human serum albumin. Molecules.

[73] Babcock J.J., Brancaleon L. (2013). Bovine serum albumin oligomers in the E- and B-forms at low protein concentration and ionic strength. Int. J. Biol. Macromol..

[74] Deguchi S., Alargova R.G., Tsujii K. (2001). Stable dispersions of fullerenes, C60 and C70, in water. Preparation and characterization. Langmuir.

[75] Bellotti E., Cascone M.G., Barbani N., Rossin D., Rastaldo R., Giachino C., Cristallini C. (2021). Targeting cancer cells overexpressing folate receptors with new terpolymer-based nanocapsules: Toward a novel targeted dna delivery system for cancer therapy. Biomedicines.

[76] Shimada R., Hino S., Yamana K., Kawasaki R., Konishi T., Ikeda A. (2022). Improvement of Photodynamic Activity by a Stable System Consisting of a C60 Derivative and Photoantenna in Liposomes. ACS Med. Chem. Lett..

[77] Kawasaki R., Antoku D., Ohdake R., Sugikawa K., Ikeda A. (2020). Bacterial elimination via the photodynamic activity of a fullerene/light-harvesting antenna molecule assembled system integrated into liposome membranes. Nanoscale Adv..

[78] Antoku D., Satake S., Mae T., Sugikawa K., Funabashi H., Kuroda A., Ikeda A. (2018). Improvement of Photodynamic Activity of Lipid–Membrane-Incorporated Fullerene Derivative by Combination with a Photo-Antenna Molecule. Chem.—A Eur. J..

[79] Liu J., Şen Karaman D., Zhang J., Rosenholm J.M., Guo X., Cai K. (2017). NIR light-activated dual-modality cancer therapy mediated by photochemical internalization of porous nanocarriers with tethered lipid bilayers. J. Mater. Chem. B.

[80] Lee S.Y., Jeon S.I., Jung S., Chung I.J., Ahn C.H. (2014). Targeted multimodal imaging modalities. Adv. Drug Deliv. Rev..

