# Supplementary material for: Enhanced Uptake and Phototoxicity of C60@albumin Hybrids by Folate Bioconjugation
Source: Nanomaterials (Basel). 2022 Oct 6;12(19):3501. doi: 10.3390/nano12193501 (PMC9565331; doi:10.3390/nano12193501)
Supplement: Supplementary file 1 [file nanomaterials-12-03501-s001.zip › nanomaterials-1906807-supplementary.pdf]

Enhanced Uptake and Phototoxicity of C_{60}@albumin Hybrids by Folate Bioconjugation

Andrea Cantelli, Marco Malferrari, Edoardo Jun Mattioli, Alessia Marconi, Giulia Mirra, Alice Soldà, Tainah Dorina Marforio, Francesco Zerbetto, Stefania Rapino *, Matteo Di Giosia *, Matteo Calvaresi *

Dipartimento di Chimica "Giacomo Ciamician", Alma Mater Studiorum-Università di Bologna, Via Francesco Selmi 2, 40126 Bologna, Italy

* Correspondence: stefania.rapino3@unibo.it (S.R.); matteo.digiosia2@unibo.it (M.D.G.); matteo.calvaresi3@unibo.it (M.C.)

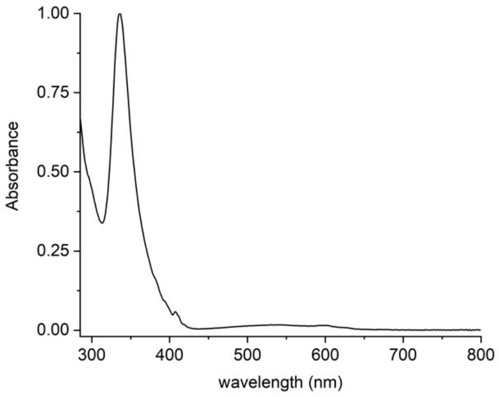

Figure S1. Normalized UV-visible spectrum of C_{60} in toluene.

Determination of the binding stoichiometry between C_{60} and HSA

We calculated the average number of C_{60} molecules bound to HSA from the UV-vis spectrum of the C_{60}@HSA adduct (Figure 1a), using the Lambert-Beer law.

Considering that:

1) The initial concentration of the HSA solution is 10^{-4} M. The UV-vis spectrum in figure 1 was obtained with a 1/50 dilution, so that the concentration of HSA in the sample is $2*10^{-6}$ M.

2) At 341 nm only C_{60} absorbs, and the C_{60}@HSA adduct has an absorbance value of 0.163

3) The molar extinction coefficients of C_{60} at 341 is 18900 $M^{-1}cm^{-1}$.

4) The concentration of C_{60} in the sample is $8.6*10^{-6}$ M.

5) An average number of 4.3 fullerenes molecules resulted bound to a single HSA.

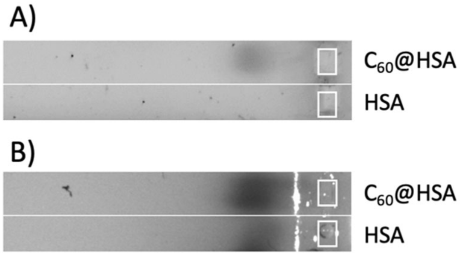

Figure S2. Agarose gel electrophoresis of HSA and C_{60}@HSA acquired in colorimetric mode. A) Before staining. B) After staining. The gel was stained with Coomassie Blue.

The visible spot of C_{60}@HSA, before staining, is due to the presence of C_{60} that absorbs in the visible region.

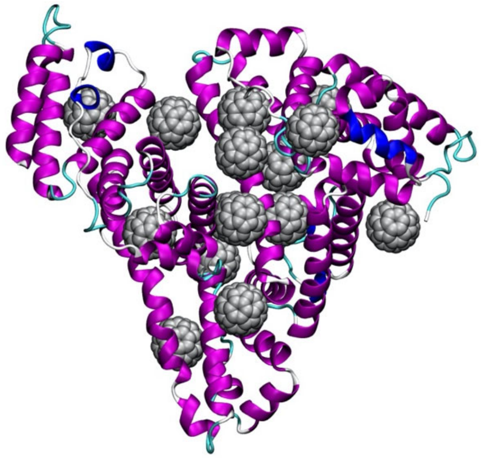

Figure S3. Possible binding site of C_{60} in HSA, identified by docking.

Binding site 1

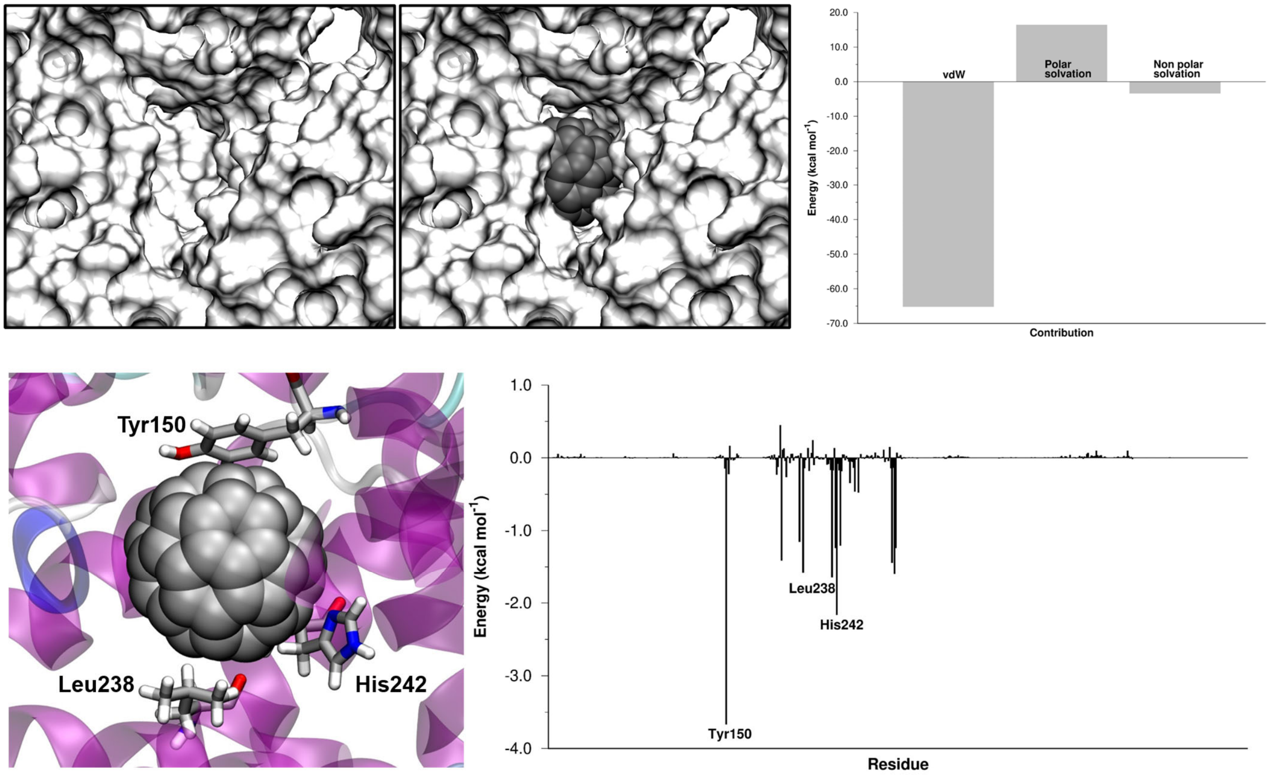

Figure S4. Interaction between HSA and C_{60} bound in the binding site 1 (Sudlow site 1 - FA7).

Top. Surface complementarity between the binding site 1 and the C_{60} cage (on the left). Energy components of $\Delta E_{binding}$ (on the right). *Bottom*. Interaction between C_{60} and the three most interacting amino acids of the binding site 1 (on the left). $\Delta E_{binding}$ decomposed per residue (on the right).

Binding site 2

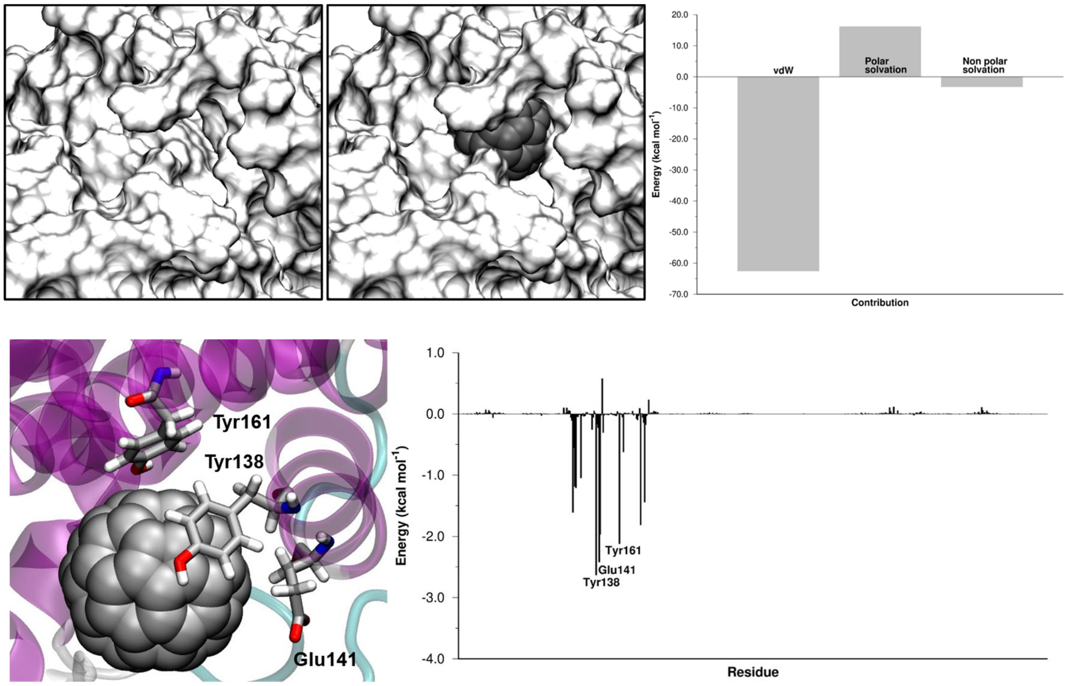

Figure S5. Interaction between HSA and C_{60} bound in the binding site 2 (heme binding pocket - FA1).

Top. Surface complementarity between the binding site 2 and the C_{60} cage (on the left). Energy components of $\Delta E_{binding}$ (on the right). *Bottom*. Interaction between C_{60} and the three most interacting amino acids of the binding site 2 (on the left). $\Delta E_{binding}$ decomposed per residue (on the right).

Binding site 3

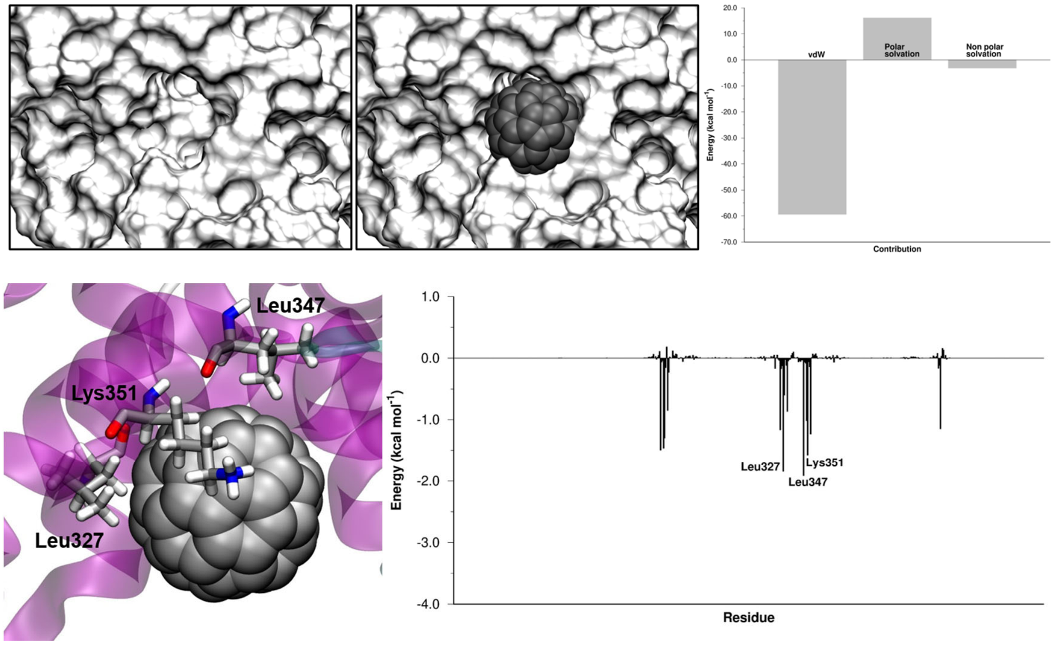

Figure S6. Interaction between HSA and C_{60} bound in the binding site 3 (albumin-binding side FA6).

Top. Surface complementarity between the binding site 3 and the C_{60} cage (on the left). Energy components of $\Delta E_{binding}$ (on the right). *Bottom*. Interaction between C_{60} and the three most interacting amino acids of the binding site 3 (on the left). $\Delta E_{binding}$ decomposed per residue (on the right).

Binding site 4

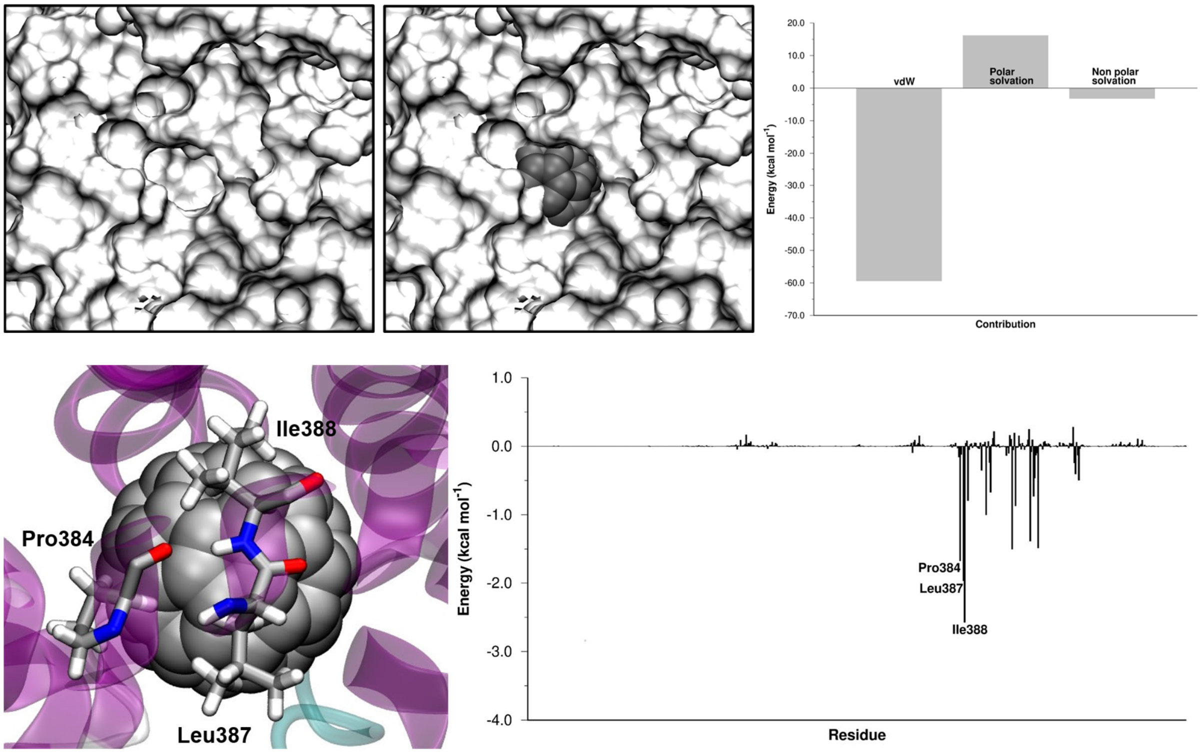

Figure S7. Interaction between HSA and C_{60} bound in the binding site 4 (Sudlow site 2 - FA3,4).

Top. Surface complementarity between the binding site 4 and the C_{60} cage (on the left). Energy components of $\Delta E_{binding}$ (on the right). *Bottom*. Interaction between C_{60} and the three most interacting amino acids of the binding site 4 (on the left). $\Delta E_{binding}$ decomposed per residue (on the right).

Binding site 5

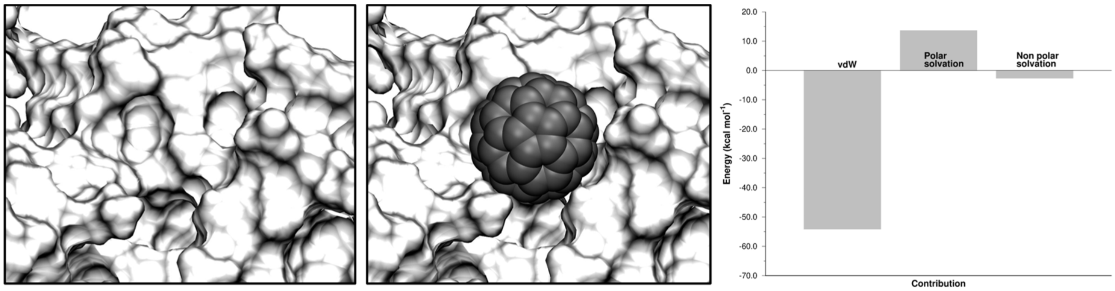

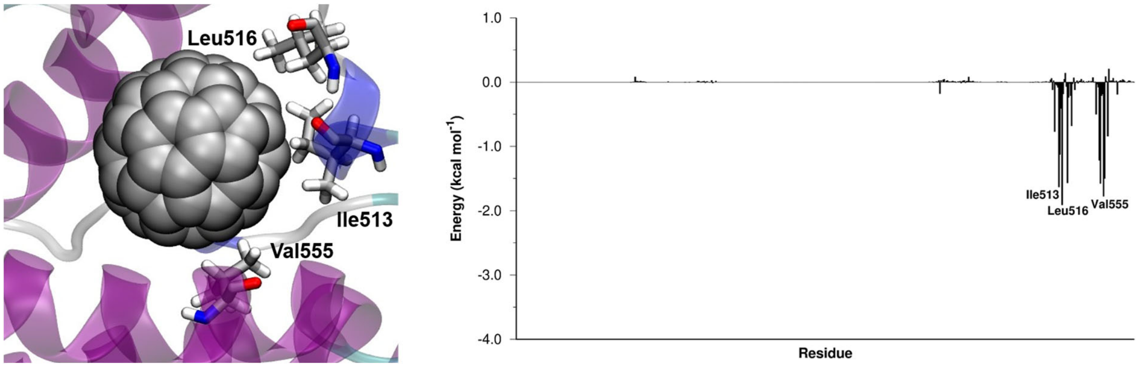

Figure S8. Interaction between HSA and C_{60} bound in the binding site 5 (albumin-binding side FA5).

Top. Surface complementarity between the binding site 5 and the C_{60} cage (on the left). Energy components of $\Delta E_{binding}$ (on the right). *Bottom*. Interaction between C_{60} and the three most interacting amino acids of the binding site 5 (on the left). $\Delta E_{binding}$ decomposed per residue (on the right).

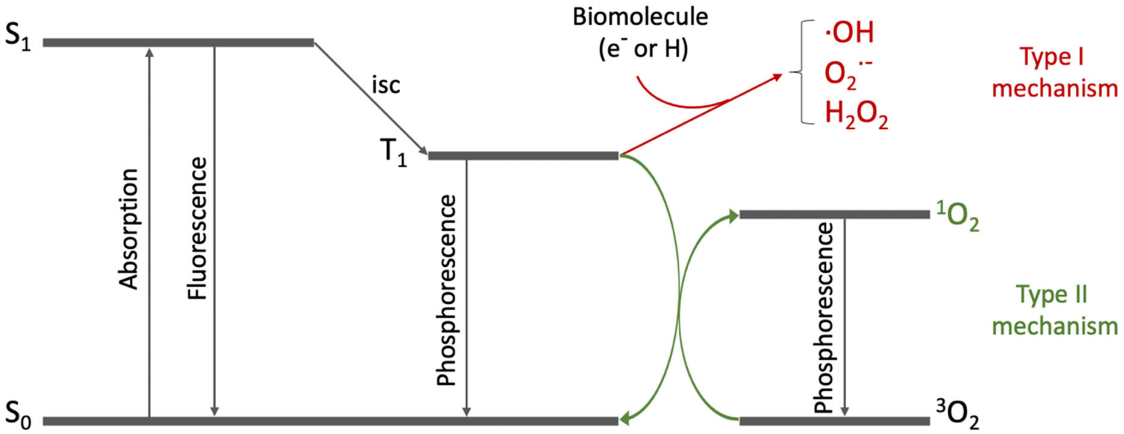

Scheme S1. Jablonski's diagram showing Type I and type II mechanism of ROS generation.

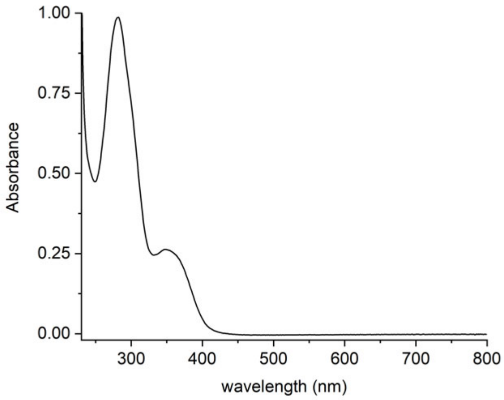

Figure S9. Normalized UV-visible spectrum of FA in PBS.

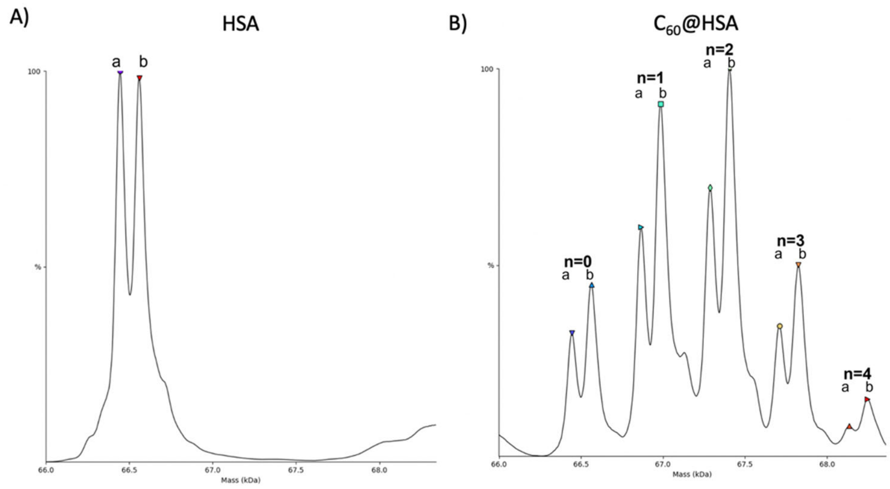

Figure S10. Mass spectra of HSA A) before and B) after conjugation with FA. A) For HSA two peaks (a,b) were observed that correlates with the calculated mass of HSA isoforms. B) The same analysis on the product of the conjugation procedure, HSA-FA, yielded peaks equal to the mass of HSA conjugated with n folate moieties.

The results of mass spectrometry analysis confirmed the covalent attachment of FA following the conjugation procedure.

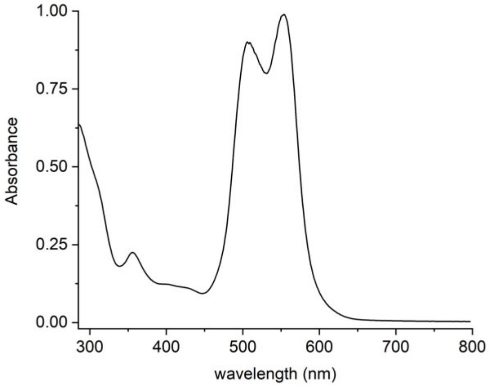

Figure S11. Normalized UV-visible spectrum of TRITC in PBS.

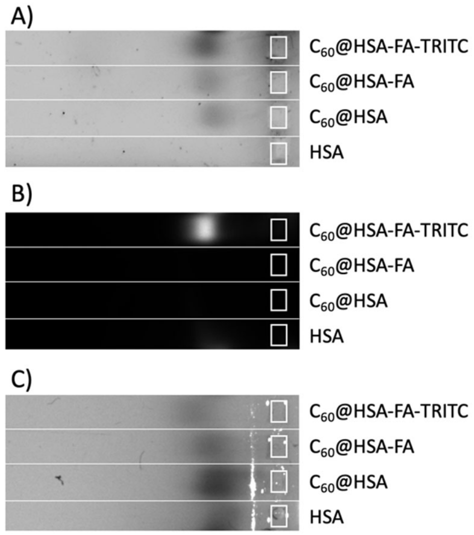

Figure S12. Agarose gel electrophoresis of HSA, C_{60}@HSA, C_{60}@HSA-FA and C_{60}@HSA-FA-TRITC A) Before staining, acquired in colorimetric mode. B) Before staining, acquired in fluorescent mode C) After staining, acquired in colorimetric mode. The gel was stained with Coomassie Blue.

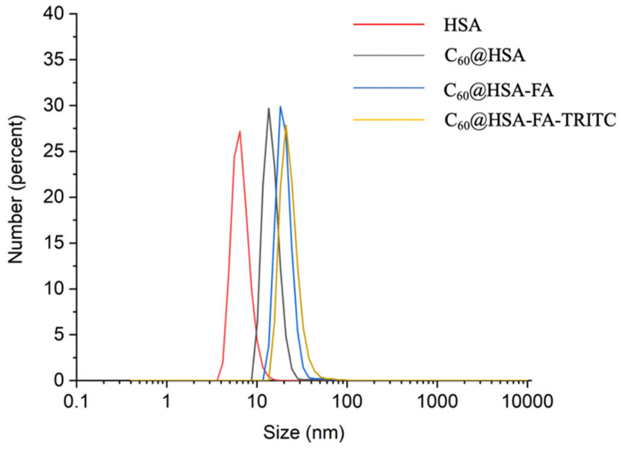

Figure S13. Particle number size distribution of HSA (red); C_{60}@HSA(grey); C_{60}@HSA-FA(blue); C_{60}@HSA(gold).

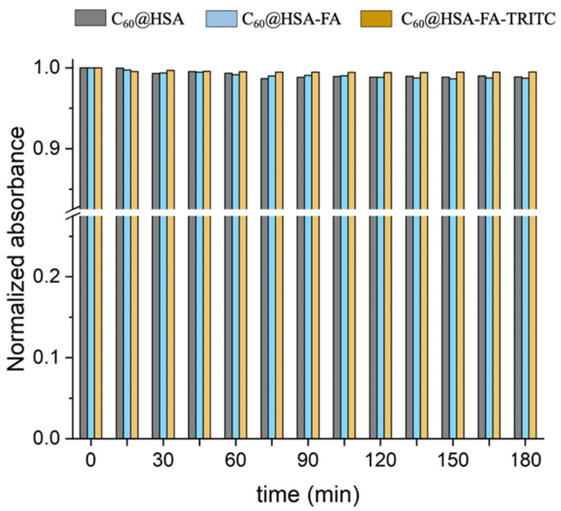

Figure S14. Evolution of the stability of C_{60}@HSA(grey); C_{60}@HSA-FA(blue); C_{60}@HSA(gold) as a function of time, in phosphate buffer saline (PBS). The absorbance of fullerene diagnostic band (341 nm) of the bioconjugates was measured. The initial value of absorbance was normalized to 1.

Table S1. – ANOVA Two-way analysis.

		Probability	Confidence level
C_{60}@HSA-FA, 18h	C_{60}@HSA-FA, 3h	0.365	No significantly different
C_{60}@HSA, 3h	C_{60}@HSA-FA, 3h	$1.47 \cdot 10^{-8}$	> 99,9%
C_{60}@HSA, 3h	C_{60}@HSA-FA, 18h	$1.51 \cdot 10^{-8}$	> 99,9%
C_{60}@HSA, 18h	C_{60}@HSA-FA, 3h	$1.47 \cdot 10^{-8}$	> 99,9%
C_{60}@HSA, 18h	C_{60}@HSA-FA, 18h	$1.51 \cdot 10^{-8}$	> 99,9%
C_{60}@HSA, 18h	C_{60}@HSA, 3h	1	No significantly different

Probability: probability that the two mean values are not significantly different.
Confidence level: percentage of significance in mean difference.
